# Defining the next generation of *Plasmodium vivax* diagnostic tests for control and elimination: Target product profiles

**DOI:** 10.1371/journal.pntd.0005516

**Published:** 2017-04-03

**Authors:** Xavier C. Ding, Maria Paz Ade, J. Kevin Baird, Qin Cheng, Jane Cunningham, Mehul Dhorda, Chris Drakeley, Ingrid Felger, Dionicia Gamboa, Matthias Harbers, Socrates Herrera, Naomi Lucchi, Alfredo Mayor, Ivo Mueller, Jetsumon Sattabongkot, Arsène Ratsimbason, Jack Richards, Marcel Tanner, Iveth J. González

**Affiliations:** 1 FIND, Geneva, Switzerland; 2 Department of Communicable Diseases and Health Analysis, Pan American Health Organization/World Health Organization, Washington, DC, Unites States of America; 3 Eijkman-Oxford Clinical Research Unit, Jakarta, Indonesia; 4 The Centre for Tropical Medicine, Nuffield Department of Medicine, University of Oxford, Oxford, United Kingdom; 5 Australian Army Malaria Institute, Brisbane, Australia; 6 Global Malaria Programme, World Health Organization, Geneva, Switzerland; 7 Centre for Tropical Medicine and Global Health, Nuffield Department of Medicine, University of Oxford, Oxford, United Kingdom; 8 World Wide Antimalarial Resistance Network, Churchill Hospital, Oxford, United Kingdom; 9 Department of Immunology and Infection, London School of Hygiene & Tropical Medicine, London, United Kingdom; 10 University of Basel, Basel, Switzerland; 11 Swiss Tropical and Public Health Institute, Basel, Switzerland; 12 Institute of Tropical Medicine Alexander von Humboldt, Universidad Peruana Cayetano Heredia, Lima, Peru; 13 Departamento de Ciencias Celulares y Moleculares, Facultad de Ciencias y Filosofía, Universidad Peruana Cayetano Heredia, Lima, Peru; 14 RIKEN Center for Life Science Technologies, Division of Genomic Technologies, Yokohama, Japan; 15 Caucaseco Scientific Research Center, Cali, Colombia; 16 Malaria Branch, Division of Parasitic Diseases and Malaria, Centers for Disease Control and Prevention, Atlanta, Georgia, United States of America; 17 ISGlobal, Barcelona Ctr. Int. Health Res. (CRESIB), Hospital Clínic-Universitat de Barcelona, Barcelona, Spain; 18 Centro de Investigação em Saúde da Manhiça (CISM), Maputo, Mozambique; 19 Population Health and Immunity Division, Walter and Eliza Hall Institute, Parkville, Australia; 20 Department of Parasites and Insect Vectors, Institut Pasteur, Paris, France; 21 Mahidol Vivax Research Unit, Faculty of Tropical Medicine, Mahidol University, Bangkok, Thailand; 22 Programme National de Lutte contre le Paludisme, Antananarivo, Madagascar; 23 University of Antananarivo, Antananarivo, Madagascar; 24 Centre for Biomedical Research, Burnet Institute, Melbourne, Victoria, Australia; 25 Department of Medicine, University of Melbourne, Parkville, Victoria, Australia; Fundaçao Oswaldo Cruz, BRAZIL

## Abstract

The global prevalence of malaria has decreased over the past fifteen years, but similar gains have not been realized against *Plasmodium vivax* because this species is less responsive to conventional malaria control interventions aimed principally at *P*. *falciparum*. Approximately half of all malaria cases outside of Africa are caused by *P*. *vivax*. This species places dormant forms in human liver that cause repeated clinical attacks without involving another mosquito bite. The diagnosis of acute patent *P*. *vivax* malaria relies primarily on light microscopy. Specific rapid diagnostic tests exist but typically perform relatively poorly compared to those for *P*. *falciparum*. Better diagnostic tests are needed for *P*. *vivax*. To guide their development, FIND, in collaboration with *P*. *vivax* experts, identified the specific diagnostic needs associated with this species and defined a series of three distinct target product profiles, each aimed at a particular diagnostic application: (i) point-of-care of acutely ill patients for clinical care purposes; (ii) point-of-care asymptomatic and otherwise sub-patent residents for public health purposes, e.g., mass screen and treat campaigns; and (iii) ultra-sensitive not point-of-care diagnosis for epidemiological research/surveillance purposes. This report presents and discusses the rationale for these *P*. *vivax-*specific diagnostic target product profiles. These contribute to the rational development of fit-for-purpose diagnostic tests suitable for the clinical management, control and elimination of *P*. *vivax* malaria.

## Author summary

*Plasmodium vivax* is the second most prevalent *Plasmodium* species amongst the five that can infect humans and cause malaria. The control and elimination of *P*. *vivax* is complicated by its specific biology, such as hard-to-detect low densities of blood-circulating parasites in infected individuals, the existence of persistent liver forms causing relapse, or the early appearance of sexual stages of the parasite during the course of an infection, which facilitates its transmission. These difficulties are reinforced by the fact that most antimalarial tools have been developed primarily for *P*. *falciparum*, the most prevalent malaria species, and are not always as effective for *P*. *vivax*. Current tools for the diagnosis of *P*. *vivax* are of limited effectiveness. Rapid diagnostic tests exist but show, in average, lower performance than similar test for *P*. *falciparum*. *P*. *vivax* diagnosis often relies on light microscopy which is challenging to maintain at a high quality and not sensitive enough to detect a large fraction of all infections. Recognizing that better diagnostic tools for *P*. *vivax* are needed, we report in this study the development of new target product profiles to define the specific characteristics of such tests. The establishment of these consensus-based documents is an important first step to guide research and development efforts toward better diagnostic solutions for *P*. *vivax* malaria and to accelerate the elimination of this species alongside *P*. *falciparum*.

## Introduction

### The need for better *Plasmodium vivax* diagnostic tests

The concerted international efforts initiated near the turn of this century to move from malaria control to malaria elimination and ultimately eradication show remarkable progress during the last decade [1]. Financial, political and scientific commitment to solve the malaria problem led so far to an overall 37% decrease in global incidence between the years 2000 and 2015 and an estimated 60% decrease in mortality during this period [1]. As a result, in sub-Saharan Africa, where most known cases of malaria occur, malaria is no longer the prime cause of death for children below the age of 5 years old. These gains have been driven by the cumulative impacts of multiple entomological and antimalarial interventions implemented via improved policies. Since 2010 the World Health Organization (WHO), for example, recommended confirmation of suspected malaria cases using rapid diagnostic test (RDT) or by the examination of a stained blood smear by light microscopy (LM). The practice of presumptive treatment without confirmation was thus discouraged. Since then, the estimated number of malaria diagnostic tests performed globally on suspected cases has risen significantly, especially in the African WHO Region where the proportion of tested cases has increased from 41% in 2010 to 65% in 2014. This increase in testing is largely due to the availability of quality-assured RDTs. The number of RDT distributed by National Malaria Control Programs rose from less than 25 millions in 2008 to more than 125 millions in 2014 in the African WHO Region, whereas it remained relatively constant in other areas where *P*. *vivax* is present [1]. This highlights the importance of high quality, affordable and easy to use point-of-care tests to facilitate the effective diagnosis and prompt treatment of malaria.

This encouraging portrait of progress does not fully extend to the malaria caused by *P*. *vivax*. In fact, *P*. *vivax* prevalence appear to decrease slower than that of *P*. *falciparum*, which results in a significant shift to *P*. *vivax* predominance almost everywhere outside sub-Saharan Africa. *P*. *vivax* is now the sole or main cause of malaria in approximately three quarters (26/34) of the endemic countries currently in the elimination phase, suggesting that this species will be much more difficult to eliminate than *P*. *falciparum*. Although approximately one half (47%) of all malaria cases outside sub-Saharan Africa are caused by *P*. *vivax* and about 2.8 billion people live at risk of infection [2], it has been neglected in science, clinical medicine and public health until very recently [3–5]. This has resulted in strategies and tools for malaria control and elimination suited to *P*. *falciparum* but not *P*. *vivax*. Specific biological traits of *P*. *vivax* explain that poor fit. First, a single infectious bite of a mosquito leads to a primary attack within about 2 weeks, but then goes on to cause multiple clinical attacks at intervals of about 2 months for as long as 4 years (but typically 2 years or less). Those later attacks, called relapses, derive from dormant liver stages of *P*. *vivax* called hypnozoites. These forms have been shown to cause at least 80% of all *P*. *vivax* blood-stage infections in Papua New Guinea [6], and 96% of attacks at the Thai-Myanmar border [7]. Second, during the course of a blood stage infection, gametocytes appear simultaneously with the same 48 hour developmental cycle as asexual parasites and, often, before the onset of symptoms and, while they do not seem to persist as long as *P*. *falciparum* gametocytes, their infectiousness to mosquitoes may be relatively higher [8–11]. Third, *P*. *vivax* merozoites invade only the most immature reticulocytes that most often occur in bone marrow rather than in circulation [12]. The bulk of *P*. *vivax* biomass may occur in extravascular tissues of the marrow and spleen rather than in circulating blood, whereas *P*. *falciparum* is largely impounded within vascular sinuses [13]. Those observations help explain why parasitaemias by *P*. *vivax* are naturally and consistently much lower compared to *P*. *falciparum*. Parasite densities of *P*. *vivax* at clinical presentation are typically in the range of 4 000 +/- 3 000 parasite per μL of blood (p/μL), which is three to four-times lower than for *P*. *falciparum*, and peak parasitaemias rarely exceeds 100 000 p/μL in *P*. *vivax* but is quite common in *P*. *falciparum* [14–18]. In fact, a large proportion of all *P*. *vivax* infections, up to 70% in certain areas, have been found to be below the limit of detection (LOD) of microscopy [19]. Although present in all settings, submicroscopic infections appear to be clearly of higher relative importance in low prevalence areas, representing an additional challenge to elimination efforts [19].

The control and elimination of *P*. *vivax* is thus more complex than with *P*. *falciparum* as it requires the rapid diagnosis of infection at lower parasite densities, but also initiating radical cure treatment for hypnozoites in conjunction with acute treatment for blood stage parasites. Problematically, the only currently available hypnozoitocidal therapy, the 8-aminoquinoline primaquine regimen, is typically 14-days in duration and exposes glucose-6-phosphate dehydrogenase (G6PD) deficient patients–a widespread genetic disorder impacting 8% of residents of malaria endemic nations [20]–to potentially life-threatening acute haemolytic anaemia. Finally, some of the natural polymorphisms in the P450 cytochrome type 2D6 (CYP2D6) result in null or impaired metabolism of primaquine to its active metabolite and cause therapeutic failure against relapse [21–23].

The increasing use of RDTs for the diagnosis of malaria resulted in significant progress in the past ten years but less so for *P*. *vivax* specifically. Relatively poor diagnostic performance of the most widely used RDTs for non-*falciparum* malaria may help explain continuing reliance upon microscopy for the diagnosis of *P*. *vivax* in endemic areas. RDTs for *P*. *vivax* are generally considered of lower accuracy, with performance, stability and false positivity issues being commonly reported in the literature [24–27]. The actual diagnostic coverage and the analytical performances of *P*. *vivax* RDTs are poorly documented. These deficiencies have been clearly recognised in the Malaria Eradication Research Agenda (malERA) initiative, which has expressed the high priority need for “more sensitive tests for *P*. *vivax* for case management” [28]. This initiative also highlighted that RDTs for *P*. *vivax* “lack consistency in sensitivity and stability” [28]. While a recent review has indicated that the quality of *P*. *vivax* RDTs is improving, only 59% (17/29) of *P*. *vivax* RDTs displayed an acceptable panel detection score (PDS) at 200 parasites per μl of blood as compared to 93% (38/41) for *P*. *falciparum* RDTs during the latest WHO-FIND product testing of malaria RDTs [25,29]. While *P*. *vivax* infections can be identified via the detection of *Plasmodium* specific aldolase or plasmodial lactate dehydrogenase (pLDH) enzymes, the univocal identification of *P*. *vivax* requires the specific detection of the pLDH isoform of this species (Pv-pLDH). As a proxy of the typical performances of this type of test, the average PDS of Pv-pLDH based RDTs appear significantly lower than that of the RDTs detecting *P*. *falciparum* specific histidine rich protein 2 (HRP2) when considering the cumulative results of the WHO-FIND Product Testing Programme (Table 1). This illustrates the shortcomings associated with current *P*. *vivax* specific RDTs.

**Table 1:** Average panel detection scores of quality-controlled RDTs from WHO-FIND Product Testing Programme (n = 126).

Species	Antigen	n	Average PDS^a^ and range
*P*. *vivax*	Pv-pLDH	32	59% (0%-100%)
*P*. *vivax*	Pvom-pLDH^b^	3	77% (63%-91%)
*P*. *vivax*	aldolase	6	41% (0%-82%)
*P*. *falciparum*	Pf-pLDH	9	52% (6%-89%)
*P*. *falciparum*	HRP2	113	82% (32%-99%)

^a^Average panel detection score (PDS) of the corresponding *Plasmodium* species at 200 parasites per μL of blood. Extracted from [30].

^b^Representing the pLDH epitopes common to *P*. *vivax*, *P*. *ovale*, and *P*. *malariae*, enabling the indiscriminate detection of these three species.

Performance of light microscopy is directly dependent on operator proficiency and sample preparation, and species determination in areas of *P*. *falciparum* and *P*. *vivax* co-endemicity may be challenging [31,32]. Microscopy, like RDTs, also suffers limited sensitivity and often fails to identify a substantial fraction of *P*. *vivax* infections of blood [19]. Alternative diagnostic methods, based on nucleic acid amplification techniques (NAATs) and serological markers exist or are emerging. While microscopy and RDTs are recommended by WHO as “the primary diagnostic tools for the confirmation and management of suspected clinical malaria in all epidemiological situations including areas of low transmission as well as for routine malaria surveillance”, a potential role for NAAT- and serology-based approaches is considered relevant in areas of low endemicity and near elimination for epidemiological research and surveys aimed at mapping submicroscopic infections to guide intervention measures specific to these settings [33]. While the research and laboratory applications of these tests is clear, their value for *P*. *vivax* infection detection and their optimal application are, however, currently unclear.

Currently available diagnostic tests for *P*. *vivax* are not optimal to address the full range of infection detection needs, from clinical case management to surveillance and elimination-oriented interventions through “surveillance-response”. Currently, poor diagnostic effectiveness contributes to the resilience of *P*. *vivax* to global and national malaria intervention strategies.

### Defining target product profiles for *Plasmodium vivax* diagnostic tests

In order to facilitate the development of improved *P*. *vivax* tests, a set of target product profiles (TPPs) addressing the specific needs associated with this species were developed through expert consensus. These TPPs are intended to guide the efforts of test developers, donors and other stakeholders in the global health community to address the *P*. *vivax* challenge. A limited number of TPPs for malaria diagnostic tests have been developed in the past few years (S1 Table). The malERA initiative published two generic TPPs in 2011, one for the diagnosis of malaria clinical cases and one for screening and surveillance activities, with parameters that could be applied to both, *P*. *falciparum* and *P*. *vivax* [28]. In 2014, the 10th session of the WHO Malaria Policy Advisory Committee Meeting released recommendations for malaria diagnosis in low transmission settings and described the ideal characteristics of future tests for this application, without defining species-specific needs [34]. A malaria diagnostic TPP was also developed by PATH for the Diagnostics for Malaria Elimination Toward Eradication (DIAMETER) project that supports the development and implementation of diagnostic solutions for malaria elimination [35]. The format and target of the test described in this profile are restricted to that of lateral flow immunoassay detecting HRP2 for *P*. *falciparum* infections. Finally, while it did not include a full TPP, the 2015 WHO technical brief about the control and elimination of *P*. *vivax* malaria highlighted the need for research to develop tests that can detect *P*. *vivax* at a minimum of 25 p/μL of blood as well as tests that “can detect submicroscopic, asymptomatic infections in elimination settings, where it is critical to detect all infections” [3]. While the very specific biological and clinical nature of *P*. *vivax* infections requires adapted tools, none of these TPPs addressed the needs of *P*. *vivax* infection detection. To fill this gap, FIND, a not-for-profit organization supporting the development and implementation of diagnostic solutions for diseases of poverty, consulted with *P*. *vivax* experts, all co-authors of this publication, to define the diagnostic needs for *P*. *vivax* and established consensus-based TPPs, with the goal to guide product development efforts toward optimized diagnostic solutions and to ultimately accelerate elimination of this malaria species.

## Methods

TTPs were developed in an iterative and consensus-decision-making process involving a large number of experts from academic research institutions, national malaria control programmes, the WHO Global Malaria Programme, and the WHO Americas Regional Office (AMRO)/Pan American Health Organization (PAHO). An initial expert meeting took place in October 2015 to review current practices and topics of interest for the diagnosis of *P*. *vivax* malaria. Three TPPs were defined based on specific intended uses, a list of forty-three TPP characteristics to be informed was established based on an initial list proposed by FIND, and preliminary values for each of these characteristics were discussed. TPPs were gradually refined through five rounds of drafts review and update through online communication (draft versions 0.1 to 0.6). Finally, an online survey was conducted to collect comments from each contributor on the remaining debated characteristics and establish a majority vote to issue final TPPs (versions 1.0) reported here.

For each TPP, the intended use, target populations and users, the implementation level as well as expected performance, operational and financial characteristics were defined. For most of these characteristics, minimal and optimal values have been defined, providing a range of values from the minimally acceptable value to the ideal one. The minimal values have been typically set to provide a distinguishing advantage over existing diagnostic solutions for *P*. *vivax* while the optimal ones were defined as the value that could provide optimal diagnostic effectiveness.

## Results

### Intended uses

**Fig 1:**
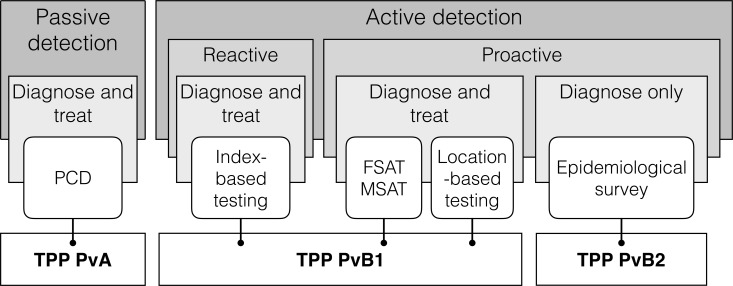
Malaria testing strategies and TPP coverage. Testing strategies are typically classified as passive and active detection where passive detection concerns symptomatic cases and active detection all infections, symptomatic or not. Passive detection is used for the confirmation of symptomatic suspected cases presenting to the healthcare system where treatment is based on a positive parasitological test (PCD: passive case detection). Active detection is typically divided as reactive and proactive detection where reactive detection consists of the active screening of a set of individuals linked geographically or sociologically to an index case for infection detection and treatment. Proactive detection can either be linked with treatment in focal screen-and-treat (FSAT) or mass screen-and-treat (MSAT) interventions or in location-based testing (*e*.*g*. boarder screening) or be independent of treatment in epidemiological surveys. The coverage of each of the three TPPs for *P*. *vivax* diagnostic tests, PvA, PvB1 and PvB2, is indicated in relation to these testing strategies. The classification of intervention types is adapted from [36].

The malaria diagnostic needs are wide and primarily defined by the type of infection to be detected, either restricted to clinical cases or including the largest possible number of infections, regardless of symptoms. Additional important factors are the test outcome, which can be to guide treatment or only to inform surveillance systems, and the implementation level, which will determine how simple to implement a given test needs to be. Three TPPs were defined to cover three distinct intended uses across this spectrum (Fig 1). TPP PvA (Pv stands for *P*. *vivax*) is addressing the diagnosis of *P*. *vivax* clinical symptomatic infection for confirmation of suspected cases (passive case detection). The two other TPPs (TPP PvB1 and PvB2) are geared toward elimination settings and address the diagnosis of all infections, symptomatic or not. TPP PvB1 addresses the need for point-of-care diagnosis of *P*. *vivax* infections regardless of the presence of symptoms including sub-microscopic parasitaemia, enabling proactive and reactive infection detection interventions, while TPP PvB2 specifically addresses the requirements for a population screening test for *P*. *vivax* infection surveillance and epidemiological surveys, independent of individual infection treatment. The specific intended uses and test outcomes as well as key distinguishing characteristics for these three TPPs are summarized in Table 2.

**Table 2:** Summary of key distinguishing features of TPP PvA, PvB1, and PvB2

Type	Characteristic	PvA	PvB1	PvB2
**Scope**	Intended use	For parasitological confirmation of symptomatic suspected cases of *P*. *vivax* malaria	For parasitological confirmation of all infections of *P*. *vivax* malaria (symptomatic and asymptomatic)	For indication of present or recent *P*. *vivax* infection for epidemiological surveys and surveillance activities
	Test outcome	Guide individual treatment in passive case detection	Guide individual treatment in reactive and proactive case detection	Inform epidemiological surveys, guide population interventions
	Target population	All individuals suspected to suffer from clinical *P*. *vivax* infection	All individuals susceptible to suffer from *P*. *vivax* infection in endemic settings	All individuals in an endemic setting
	Target users	M^b^: Community and facility-based health workers	M: Community and facility-based health workers	M: Laboratory technicians
	Implementation level	Community health facilities, health posts, health centers	Community health facilities, health posts, health centers	District hospitals and reference laboratories
**Performance**	Analytical sensitivity^a^	M: 25 p/μL O^b^: 5 p/μL	M: 20 p/μL O: 1 p/μL	M: 0.1 p/μL (irrelevant for recent past infection detection) O: 0.01 p/μL (irrelevant for recent past infection detection)
	Analytical specificity	M: Discriminate *P*. *vivax* form other *Plasmodium spp*. O: Discriminate between *P*. *vivax*, *P*. *falciparum* and other *Plasmodium spp*. No cross-reactivity with other pathogens	M: Discriminate *P*. *vivax* from other *Plasmodium spp*. O: Discriminate between *P*. *vivax*, *P*. *falciparum* and other *Plasmodium spp*. No cross-reactivity with other pathogens	M: Discriminate between *P*. *vivax*, *P*. *falciparum* and other *Plasmodium spp*.. O: Discriminate between all *Plasmodium spp*. No cross-reactivity with other pathogens
	Diagnostic sensitivity^c^	M: >95% O: ≥ 99%	M: >95% O: ≥ 99%	M: >95% O: ≥ 99%
	Diagnostic specificity^c^	M: >95% O: ≥ 99%	M: >95% O: ≥ 99%	M: >95% O: ≥ 99%
**Operational aspects**	Assay format	M and O: Single-use *in vitro* diagnostic	M and O: Single-use *in vitro* diagnostic	M: 96-well format O: 384-well format
	Assay throughput	Single assessment per test	Single assessment per test with the option to batch test up to 100 samples per run in a POC format	Batch testing in line with assay format
	Equipment	M: small (<100 cm^2^ footprint) and portable (<5 kg) O: none	M: small (<100 cm^2^ footprint) and portable (<5 kg) O: none	M: Transportable (<20 kg) O: Portable (<5 kg)
	Sample type	M: Capillary blood O. Capillary blood or any less invasive validated sample	M: Capillary blood O. Capillary blood or any less invasive validated sample	M: Capillary blood O. Capillary blood or any less invasive validated sample
	Sample volume (if capillary blood)	M: ≤ 100 μL O: ≤ 50 μL	M: ≤ 100 μL O: ≤ 50 μL	M: ≤ 200 μL O: ≤ 100 μL
	Time-to-result	M: ≤ 1 hour O: ≤ 30 minutes	M: ≤ 6 hours O: ≤ 30 minutes	M: ≤ 1 month O: ≤ 7 days
**Cost**	End user price per test	M: ≤1.0 USD O: ≤0.5 USD	M: ≤2.0 USD O: ≤1.0 USD	M: ≤1.0 USD O: ≤0.1 USD
	Cost of diagnosis per sample	M: ≤2.0 USD O: ≤1.0 USD	M: ≤5.0 USD O: ≤2.0 USD	M: ≤1.2 USD O: ≤0.5 USD

^a^Values in parasite per μL of blood might not be relevant for all assay types, especially for TPP PvB2, which is not for a parasitological test and includes the detection of recent infection.

^b^M: minimal, O: optimal

^c^as compared to standard PCR with a know limit of detection of 1 p/μL (PvA and PvB1) and a method with an analytical sensitivity at least equal to that of the index test (PvB2).

### TPP PvA: Diagnosis of *P*. *vivax* malaria acute infection

TPP PvA addresses the need for better diagnostic for the parasitological confirmation of clinical cases in passive case detection scenarios (S2 Table). This TPP is therefore designed for a point-of-care test that is simple to implement (requiring ideally half-a-day of training and three steps or less) and rapid (time-to-results ≤ 30 min.) to guide prompt clinical management of *P*. *vivax* malaria patient: blood-stage treatment of acute *P*. *vivax* infections as well as radical cure for populations to which 8-aminoquinolines can be administrated safely.

Current tests for this intended use are RDTs and microscopy and the characteristics of this TPP were established with the objective to overcome the limitations of these tests. A key performance characteristic for this TPP is the analytical sensitivity. Expert microscopy is considered to provide a LOD of 50 p/μL but this value is typically assumed to be significantly higher in many endemic areas [37,38]. A recent analysis of the analytical performances of the best-in-class Pv-pLDH RDTs indicated these would fail to detect a majority of samples containing 200 p/μL (Jimenez *et al*., submitted elsewhere). A minimal target LOD of 25 p/μL would therefore represent at least a two-fold improvement over the practical microscopy LOD and be a significant improvement over current RDTs. However, an optimal LOD should be equal or inferior to 5 p/μL, corresponding to one order of magnitude below the typical lowest peripheral parasitaemia at presentation for uncomplicated *P*. *vivax* malaria, ensuring that no clinical cases would be missed because of inadequate LOD [15,39]. Regarding diagnostic specificity, the univocal identification of *P*. *vivax* as the *Plasmodium* infecting species is essential as only this species and the relatively rare *P*. *ovale* require radical cure for liver-stage parasite removal. For areas of co-endemicity between *P*. *vivax* and *P*. *falciparum* (39 out 98 malaria endemic countries [1]), a distinguishing advantage would be the capacity to identify and discriminate between these two major species. Regarding both the diagnostic sensitivity and specificity, the minimal values have been set to at least match that of current *P*. *falciparum* RDTs and the optimal ones to provide a distinguishing advantage at 95% and 99%, respectively [25].

In terms of operational characteristics and beyond the required simplicity and rapidness of the test, stability during transport, storage and usage is important. An analysis of the typical RDT supply chain revealed that these are frequently exposed to temperature above 30°C and sometimes up to more than 40°C, hence a test destined to the same intended use needs to withstand such harsh conditions, being ideally stable for up to 12 months at 45°C and 90% relative humidity and usable at temperatures as low as 5°C and as high as 45°C.

Another crucial element often difficult to resolve is that of cost. The cost of diagnosis (including sample collection, processing, and transmission of the results to the patient) for RDT and light microscopy were evaluated to be between 1.0 and 2.0 USD in 2011 in Uganda, a *P*. *falciparum* endemic country [40]. It is somewhat complex to define at what end-user price and overall diagnosis cost a PvA test might become cost-effective as the cost of misdiagnosed and relapsing *P*. *vivax* infections is difficult to evaluate but it was assumed that the end user price should ideally not be superior to the current price of RDT (~0.5 USD) and that the overall cost of diagnosis should not be superior to the values mentioned here above.

### TPP PvB1: Point-of-care diagnosis of sub-clinical *P*. *vivax* infection

TPP PvB1 extends the scope of PvA to address the detection of all blood-stage infections, regardless of the presence of symptoms, to enable reactive and proactive case detection and treatment (S3 Table). This TPP is defining the characteristics of tests that could be deployed in elimination settings to identify the asymptomatic reservoir known to contribute to residual transmission and guide blood-stage and, if appropriate, liver-stage treatments for the asymptomatic carriers.

Similar to PvA, PvB1 tests need to be deployable in a point-of-care manner (or “point-of-contact” since it would not necessarily be used in a medical care context) and therefore require very similar characteristics in terms of ease-of-training, ease-of-use, short time-to-results and operational robustness. There is no such test currently in use and while NAATs might meet many of the required characteristics, they are not easily deployable as a point-of-care diagnostic solution.

The main distinguishing feature of PvB1 as compared to PvA is the lowered analytical sensitivity needed to (i) detect a substantial fraction of the asymptomatic and low parasitaemia infections, and thus (ii) support elimination interventions by providing crucial information about these parasite populations. A modelling study investigating the case of a *P*. *falciparum* diagnostic test used to trigger focal mass drug administration (focal MDA, *i*.*e*. village-based mass drug administration in case of a local prevalence identified above a certain threshold) suggested that in such low prevalence settings, an analytical sensitivity of 20 p/μL might suffice to ultimately reduce the parasite prevalence to zero within a ten-year time frame [41]. It is however not clear how such a model could apply to *P*. *vivax*, for which a majority of infections are relapses from liver stage parasites. A recent study evaluating the parasitaemia distribution in more than 1,500 *P*. *vivax* infected asymptomatic individuals at the Thai-Myanmar border revealed a geometric mean parasitaemia of 5.6 p/μL and a unimodal log normal distribution of parasitaemia in this population [42]. The minimally acceptable and ideal analytical sensitivity values for PvB1 tests were established around these estimates at 20 p/μL and 1 p/μL, respectively. The optimal value would allow to detect up to 58% of all asymptomatic infections according to the modelling of the data from Imwong *et al*. [42]. The other performance and operational characteristics are essentially identical between PvA and PvB1, with the notable exception that the test format of PvB1 should ideally be amenable to batch testing of up to 100 individuals relatively easily. This is in consideration of the reactive or proactive case detection scenarios for which this type of test would be used, requiring the rapid diagnosis and treatment of a potentially large number of individuals as opposed to passive case detection, where testing is normally performed on demand and as suspected cases present at health posts and medical centers.

Another key distinguishing feature of PvB1 is the cost. As mentioned above, the cost elements of diagnoses are difficult to factor in the absence of comprehensive costing analyses and cost-effectiveness studies of existing solutions. In the case of PvB1, it was assumed that the increase in analytical sensitivity requirement would translate in an increased end-user test price (minimal: 2.0 USD, optimal: 1.0 USD) and cost of diagnosis (minimal: 5.0 USD, optimal: 2.0 USD) but that these values should ideally be lower than current NAAT tests, estimated between 2 to 4 USD per reaction, excluding capital cost [43–45]. Another element typically weighted against the cost of a diagnostic test is that of a treatment course, especially when considering mass interventions. If a diagnostic test is not significantly cheaper than a treatment course, typically targeted to 1 USD or less [46], it is, from a pure economic point-of-view, cheaper to treat in the absence of testing than screen-and-treat. We dismiss this argument as too simplistic and are of the opinion that the true financial and societal costs of MDA or mass screen-and-treat cannot be distilled down to the only cost of the commodities associated with these interventions. We would not recommend for a test to be cheaper than a treatment course in order to be an adequate PvB1 diagnostic solution. This is especially true in the case of *P*. *vivax*, which requires not only blood-stage detection, but also potentially G6PD testing for radical cure.

### TPP PvB2: Population screening for *P*. *vivax* infection surveillance

The TPP PvB2 is designed to answer the needs for high quality tests for epidemiological surveillance activities (S4 Table). This TPP is similar to PvB1 in the sense that it aims to detect all infections, including asymptomatic and low parasitaemia typically not seen by RDTs or microscopy, but it differs from PvB1 in that the diagnostic outcome is not directly linked with treatment interventions at the individual level. A PvB2 test is designed to inform surveillance system and to support epidemiological surveys. Because of this nature, such a test would not need to be deployed in a point-of-care manner but would be restricted to district hospitals and national reference laboratories, and would need to provide a very high analytical sensitivity. Current tests in this category include highly complex and specialized NAAT protocols, such as high volume quantitative PCR, reverse-transcription quantitative PCR, or PCR targeting highly repetitive elements, which all reach analytical sensitivity of approximately 0.02 p/μL [47–49]. The PvB2 minimal and optimal analytical sensitivity values were set at 0.1 p/μL (one order of magnitude lower than optimal PvB1 test) and 0.01 p/μL (two-fold lower than current state-of-the art technologies), respectively. Such values would in principle allow to detect up to 80% and 93% all of *P*. *vivax* asymptomatic infection as modelled by Imwong *et al*. [42]. When defining the optimal test sensitivity, it is pivotal to take into account the sampling procedures and blood sample volume. The blood volume equivalent that is added to a molecular assay critically determines the detection of low parasitaemias. All tests that target asymptomatic individuals should thus aim at maximizing the input material. For surveillance, collecting finger prick blood samples is considered feasible, whereas larger venous samples may be collected for research purposes only. In remote settings, sampling on filter paper will be required for storage and transport. This will compromise the detection of low parasitaemia in a significant way, as filter paper punches (directly added to the reaction or extracted) can only hold a limited blood volume. Ideally, 200 μL whole blood should be used for preparation of nucleic acids, and the final DNA solution should be concentrated as much as possible. Multi-copy target genes or reverse transcription reactions can help to detect a single parasite in the large blood volume sampled [48,49].

As a surveillance tool, PvB2 also includes tests that might not necessarily detect currently occurring infection but also recent past infections, such as serological tests, as a most effective way of estimating the residual transmission in an area of interest and potentially detect hypnozoite carriers. Obviously in such cases, an analytical sensitivity expressed in parasites per μL of blood becomes irrelevant and test-specific values would have to be defined (*e*.*g*. antibody level detected by a serology test). Ideally, the analytical specificity would also be expected to be greater than that required for PvA and PvB1 tests, and the optimal specificity would be a detection and discrimination of all five *Plasmodium spp*. infecting humans. Regarding the diagnostic sensitivity and specificity, these values were defined as similar to PvA and PvB1, however the actual reference test against which they would be determined might not easily be defined since in terms of pure analytical sensitivity, an optimal PvB2 index test is likely to be the new best standard of truth. The operational characteristics for the PvB2 TPP allow for less environment-resistant tests compared to PvA and PvB1: transport, storage and operation conditions allow for cold transportation and storage and are generally set to correspond to the typical conditions found in air-conditioned reference laboratories. The assay format is at a minimum a 96-well format and ideally a 384-well format to enable high throughput and the characterization of a high number of samples concurrently. This is linked with less stringent requirement in terms of equipment, with a tolerance for electricity requirement, up to 20 kg of equipment, and sample processing, with up to 20 steps for the assay procedure being acceptable. Similarly, the time-to-result is not of the essence in this case with an optimal time-to-results being less than 7 days, but at a minimum requirement of up to one month. These characteristics might become more critical if the test is used to target focal MDA, in which the optimal value (one week) would be a requirement.

The cost associated with such a test is arguably expected to be high, since it is likely to involve complex laboratory procedures, reagents, and equipment as well as specialized and highly trained laboratory officers. Yet, the high throughput and high volume of testing required for population surveillance interventions is expected to drive the cost of such test down to an acceptable 1.0 USD end-user price per sample (minimal requirement). The optimal value was set one order of magnitude below this, at 0.1 USD, which might be achievable for a test relying on simpler procedure and limiting the sample processing steps.

## Discussion

We present here the first consensus TPPs for *P*. *vivax* specific diagnostic tests to address the particular needs for the control and elimination of malaria due to this species. A summary of key characteristics is given in the main text (Table 2) and complete TPPs are specified in the supplementary material section (S2 to S4 Tables). Consensus-based review and discussion exercises of a large group of experts in the field guided the development of key characteristics. In this report, we describe important biological specificities of *P*. *vivax* and outline in a detailed manner the diagnostic needs associated with this malaria species. TPPs have been deliberately developed in a platform and technology-independent manner so that the capacity of diagnostic solutions based on any existing or new technology to fit with these profiles can be evaluated. These include (i) TPPs for the parasitological confirmation of suspected cases in clinical settings (PvA), (ii) the detection of a large number of infections, regardless of symptoms for reactive and proactive interventions (PvB1) and (iii) the identification of the majority of current or recent past infections for population-based “surveillance-response”, *i*.*e*. an active/passive surveillance strategy that calls for response packages tailored to the respective endemic settings (PvB2). *P*. vivax, on average, has lower parasite densities than *P*. *falciparum*, placing an extra challenge on the analytical sensitivity requirements of *P*. *vivax* diagnostic tests. Another key characteristic of *P*. *vivax* is the existence of hypnozoites, which requires complex and potentially toxic treatment to be deployed to achieve radical cure. The haemolytic toxicity risk associated with primaquine requires a G6PD test to rule out significant deficiency in this enzyme before initiating a treatment course. G6PD testing should be an integral component of the management of *P*. *vivax* infection, and accurate tests to detect its deficiency are required. TPPs for G6PD deficiency testing have been previously defined by a group of experts and are publicly available [50]. The need for better G6PD tests has been previously recognized and a new point-of-care test is showing promising results [51–54]. With the development of improved G6PD tests, the opportunity of combination test to concomitantly detect *P*. *vivax* blood-stage infections and determine the G6PD status of the host should be considered from a technological and cost-effective point-of-view.

The persistence and reactivation of hypnozoites is one of the main drivers of the resilience of *P*. *vivax* to antimalarial interventions. Developing a specific hypnozoite test is widely regarded as an extremely challenging technical task. It would require the ability of a test to detect a very small number of metabolically inactive, or poorly active parasites, sequestered in the liver. Despite the theoretical advances of such a test, the group did not consider such a test to be crucial in achieving elimination as indirect diagnostic approaches might be more promising. This is because a *P*. *vivax* blood stage infections can be assumed with a high probability to be also associated with residual hypnozoite infections. Hence, the detection of low blood-stage parasitaemia (PvB1) together with the potential detection of past recent infections (PvB2) would appear to be a more promising avenue to identify most hypnozoite carriers. Alternatively the likelihood that hypnozoites would ultimately lead to new blood stage infections suggests that it might be sufficient to sustain diagnostic efforts to detect blood stage parasites and guide the administration of radical cure for the eventual removal of hypnozoites from all carriers. This approach would however require intense, long-lasting and costly surveillance efforts to be implemented and might require to specifically target the population most at risk of *P*. *vivax* infection.

The TPPs defined here are considered to be evolving documents by nature and should stimulate discussions within the malaria community and be periodically revised to ensure that they still represent the actual needs for the diagnosis of *P*. *vivax*. These profiles will also serve to evaluate current and future tests, and should facilitate an ongoing discussion on the capacity of these tests to address the existing *P*. *vivax* diagnostics gaps. They will also serve to guide innovation and development efforts in areas of greatest need. The first one of these being better tests for the clinical management of *P*. *vivax* infections, so that the positive impact that has been observed for affordable high-quality *P*. *falciparum* RDTs can also be obtained for *P*. *vivax* malaria. Second, highly sensitive point-of-care tests will be needed to support reactive and proactive infection detection strategies through the accurate detection of asymptomatic and low density parasitaemia for those settings where plain MDA will not be adequate, that is in areas of low prevalence, where overtreatment would be unacceptably high, or in areas with a high prevalence of G6PD deficiency. Finally, no control or elimination programme can bypass the need for high quality and highly sensitive prevalence survey data. While high-performance but also high-cost and high technology NAAT approaches exist, cheaper and more standardized solutions are needed.

The limitations of the TPPs are directly linked with those of our knowledge on *P*. *vivax* malaria. For instance, more research is clearly needed on the epidemiology of asymptomatic *P*. *vivax* infections. While this parasite population has been described in South East-Asia [42], similar quantitative investigations are lacking in other settings. Similarly, a better understanding of the prevalence and distribution of *P*. *vivax* in sub-Saharan Africa [55], a place from which this species was historically thought to be mostly absent, is needed. Research is also needed for the potential identification of serological biomarker which could facilitate the detection of recent past infections. In addition, cost-effectiveness studies of current diagnostics and interventions are required to guide improvements and definition of parameter to measure impact.

Since more international organizations are becoming aware of the significant and specific role played by *P*. *vivax* malaria, we think that this initial set of *P*. *vivax* specific diagnostic TPPs highlights particular diagnostic gaps and defines specific parameters that may guide the global health community to address these needs coherently and thus contribute to the ongoing successful control and elimination efforts.

### Disclaimer

The views and opinions expressed in this article are consensus views and opinions from all individual authors and do not necessarily reflect the official policy or position of any of the authors institutions.

## Supporting information

S1 Table Summary of existing malaria diagnostic TPPs. (PDF)

S2 Table TPP PvA: Diagnosis of *Plasmodium vivax* malaria acute infection. (PDF)

S3 Table TPP PvB1: Point-of-care diagnosis of subpatent *Plasmodium vivax* infection. (PDF)

S4 Table TPP PvB2: Population screening for *Plasmodium vivax* infection surveillance. (PDF)
