# Supplementary material for: Defining the next generation of Plasmodium vivax diagnostic tests for control and elimination: Target product profiles
Source: PLoS Negl Trop Dis. 2017 Apr 3;11(4):e0005516. doi: 10.1371/journal.pntd.0005516 (PMC5391123; doi:10.1371/journal.pntd.0005516)
Supplement: S1 Table — (PDF) [file pntd.0005516.s001.pdf]

S1 Table. Summary of existing malaria diagnostic TPPs

Four TPPs for malaria diagnostics have been made publicly available from various organizations over the past 5 years. Two TPPs, for case management and screening and surveillance were published by the The Malaria Eradication Research Agenda (malERA) initiative [1]. Preferred characteristics for new technologies are presented in the meeting report of WHO Evidence Review Group on Malaria Diagnosis in Low Transmission Settings [2]. Finally PATH published a draft TPP for active case detection of sub-patent and asymptomatic malaria infections within the frame of the DIAMETER project [3]. The malERA and WHO TPPs are relevant for both *P. falciparum* and *P. vivax* while the PATH DIAMETER TPP is specifically designed for *P. falciparum*.

In addition to these four TPPs, recommendations for new *P. vivax* diagnostics are briefly discussed in the recently released WHO technical brief on the Control and Elimination of *Plasmodium Vivax* Malaria [4]. These are for diagnostic methods that more readily detect parasites in a clinical setting with a sensitivity of 25 p/µL for blood stage *P. vivax* infections as well as diagnostic methods that can detect hypnozoite (liver stage *P. vivax* infections).

	**malERA: Diagnoses and Diagnostics (January 2011) [1]**		**MPAC Session 10 (March 2014) [2]**	**DIAMETER Project (March 2014) [3]**
**Intended use**	Case Management in Elimination Settings	Screening/Surveillance (District Level or Below)	Detection of parasites in low transmission settings	« Active infection detection interventions aimed at low-density and subclinical infection detection. »
**Target analyte**	*n/a*	*n/a*	*n/a*	M[a]: HRP2<br>O[a]: HRP2, one other P. falciparum specific antigen, plus a Pan or Plasmodium vivax antigen
**Analytical sensitivity**	M: ≤ 200 p/µL<br>O: < 5 p/µL	M ≤ 20 p/µL<br>O: ≤ 5 p/µL	≤ 2 p/µL	M: ≤ 10 p/µL<br>O: ≤ 5 p/µL
**Analytical specificity**	M: *P. falciparum*<br>O: *P. falciparum* / Pan<br>Negative for all pathogens and common blood disorders	M: *P. falciparum*<br>O: Discriminate all human infection Plasmodium spp., identify hypnozoite and optionally gametocyte<br>Negative for all pathogens and common blood disorders	Detection at genus level with subsequent species differentiation	M: *P. falciparum*<br>O: *P.* falciparum / *P. vivax* / *Plasmodium spp.*

	malERA: Diagnoses and Diagnostics (January 2011) [1]		MPAC Session 10 (March 2014) [2]	DIAMETER Project (March 2014) [3]
**Diagnostic sensitivity**	M: > 95%<br>O: ≥ 99%	M: > 95%<br>O: ≥ 99%	≥ 95% for *Plasmodium* genus	M: ≥ 97%<br>O: ≥ 99%
**Diagnostic specificity**	M: > 90%<br>O: > 95%	M: > 95%<br>O: > 99%	*n/a*	M: ≥ 90%<br>O: ≥ 99%
**Repeatability (inter-operators)**	Kappa > 0.9	Kappa > 0.9	*n/a*	*n/a*
**Operating conditions**	*n/a*	*n/a*	*n/a*	M: 20 to 35°C<br>O: 10 to 40°C
**Sample type and volume**	M: capillary blood from finger prick<br>O: non-invasive	M: capillary blood from finger prick<br>O: non-invasive	≤ 50 µL of blood	M: ≤ 50 µL of capillary blood from finger prick<br>O: ≤ 25 µL of less invasive sample
**Assay throughput**	*n/a*	*n/a*	48 samples / person / platform / day	M: 7 samples / person / platform / hour<br>O: >10 samples / person / platform / hour
**Time-to-result**	M: > 30 minutes<br>O: > 15 minutes	M: > 2 days<br>O: > half-day	M: > 24 hours<br>O: > 16 hours	M: > 30 minutes<br>O: > 15 minutes
**Assay format**	Usable at community level<br>Simple, few steps	Usable by medical technician	Automated end-point format, instrument independent<br>Objective reading, easy to interpret<br>Minimum number of steps	Lateral-flow immuno-chromatographic strip in cassette format.<br>High-contrast, clear results for naked-eye<br>Two (M) or no (O) timed step
**Packaging**	Individual, moisture-proof, with all required consumable included and temperature violation detection	Moisture-proof, with all required consumable included and temperature violation detection	*n/a*	O: temperature violation detection
**Reagents**	All necessary reagent provided	All necessary reagent provided	*n/a*	All necessary reagent provided
**Equipment**	All necessary equipment provided	*n/a*	Portable	Highly portable<br>Battery-powered reader if required<br>No water need
**Power requirement**	No external power source	*n/a*	*n/a*	No external power source

	malERA: Diagnoses and Diagnostics (January 2011) [1]		MPAC Session 10 (March 2014) [2]	DIAMETER Project (March 2014) [3]
**Transport and storage stability**	M: stable at > 35°C for 2 years (incl. 18 months in country)<br>O: stable at > 45°C for 36 months	M: stable at ≤ 30°C for 1 year (incl. 6 months in country)<br>O: stable at ≤ 30°C for 2 years (incl. short period at 45°C)	No transport required below 4°C<br>Stable at 4°C for > 1 year, at RT for > 6 months	M: stable at 2 - 30 °C for 18 months (+ 2 weeks at 40 °C)<br>O: stable at 2 - 40 °C for 36 months (+ 2 weeks at 50 °C)
**Control**	*n/a*	*n/a*	*n/a*	Internal control line<br>Compatible with positive control well
**Connectivity**	*n/a*	*n/a*	Desirable network connectivity for data transfer	O: Cloud-based, real-time data handling
**Training**	Half day for community health worker	≤ 1 week for pretrained medical technician	≤ 1 week	M: < 1 day<br>O: < half-day
**Biosafety**	High blood safety<br>Non-toxic reagents	High blood safety<br>Non-toxic reagents	Low contamination risk<br>No hazardous waste	High blood safety<br>No hazardous waste
**Cost**	≤ 1 USD / test	≤ 1 USD / test	Affordable	M: ≤ 2 USD / test, no capital cost except for reader<br>O: ≤ 1 USD / test, no capital cost
**Comments**	“The sensitivity required for *P. vivax* is generally at least that required for *P. falciparum*, and the parameters here should be applied to both.”		Analytical sensitivity setting based on one log improvement over expert microscopy to be a "significant improvement"	

[a]M: minimal, O: optimal

Supplementary References

1. The malERA Consultative Group on Diagnoses and Diagnostics. A Research Agenda for Malaria Eradication: Diagnoses and Diagnostics. PLoS Med. 2011;8: e1000396. doi:10.1371/journal.pmed.1000396.t001

2. World Health Organization. WHO Evidence Review Group on Malaria Diagnosis in Low Transmission Settings [Internet]. 21 Mar 2014 [cited 11 Oct 2015] pp. 1–33. Available: http://www.who.int/malaria/mpac/mpac_mar2014_diagnosis_low_transmission_settings_report.pdf

3. PATH. Target Product Profile: Point-of-Care Malaria Infection Detection Test. In: sites.path.org [Internet]. [cited 25 Jun 2016]. Available: http://sites.path.org/dx/files/2012/11/DIAMETER_IDT_TPP_FINAL_forwebsite.pdf

4. World Health Organization. Control and Elimination of Plasmodium Vivax Malaria. 2015.
