# Supplementary material for: Defining the next generation of Plasmodium vivax diagnostic tests for control and elimination: Target product profiles
Source: PLoS Negl Trop Dis. 2017 Apr 3;11(4):e0005516. doi: 10.1371/journal.pntd.0005516 (PMC5391123; doi:10.1371/journal.pntd.0005516)
Supplement: S2 Table — (PDF) [file pntd.0005516.s002.pdf]

S2 Table. TPP PvA: Diagnosis of *Plasmodium vivax* malaria acute infection

Type	Characteristic	Minimal (M) / Description	Optimal (O)	Comment
**Scope**	Intended use	The test goal is to provide a parasitological confirmation of suspected symptomatic episodes of *P. vivax* malaria to guide the management of clinical cases. Therefore, the test needs to accurately detect biologically active erythrocytic forms of *P. vivax*.		
	Test outcome	Guide blood-stage and, if appropriate, liver-stage treatment		
	Target population	The target population is any individual suspected to suffer from a symptomatic episode of *P. vivax* malaria, including neonates, children, and pregnant women.		
	Target users	The target users include community health workers with minimal training and any health worker with a similar or superior training level.		
	Implementation level	The target implementation levels are community health facilities, health posts, and health centers [5].		
**Performance**	Analytical sensitivity	Limit of detection for target analyte corresponding to a peripheral parasitaemia of 25 p/µL	Limit of detection for target analyte corresponding to a peripheral parasitaemia of 5 p/µL	"M" corresponds to typical *P. vivax* pyrogenic threshold and is in line with malERA recommendation. "O" corresponds to the order of magnitude of the lowest peripheral parasitaemia at presentation for uncomplicated *P. vivax* malaria and is more stringent than WHO recommendation (25 p/µL) but same as malERA recommendation (5 p/µL) [1,4,6-8].
	Analytical specificity	Discriminate between *P. vivax* and other *Plasmodium spp*. Do not cross-react with any other pathogen infecting humans	Discriminate between *P. vivax*, *P. falciparum* and other *Plasmodium spp*. Do not cross-react with any other pathogen infecting humans	"M" enables the specific identification of *P. vivax*. "O" provides a unique test for *P. vivax* and *P. falciparum* co-endemicity areas. Cross-reactivity between *P. vivax* and *P. ovale* might be beneficial to identify both of these relapsing species.
	Diagnostic outcome	Binary	Binary	A continuous (quantitative) outcome is not required for the intended use of the test.
	Diagnostic sensitivity	> 95% as compared to standard PCR with known limit of detection of 1p/µL	≥ 99% as compared to standard PCR with known limit of detection of 1 p/µL	"M" equals existing best *P. vivax* RDTs sensitivity (95% [0.95CI: 86% to 99%]). "O" provides a distinguishing advantage over this value (≥ upper 0.95CI) [9].

Type	Characteristic	Minimal (M) / Description	Optimal (O)	Comment
	Diagnostic specificity	> 95% as compared to standard PCR with known limit of detection of 1p/µL	≥ 99% as compared to standard PCR with known limit of detection of 1p/µL	"O" is equivalent to existing best *P. vivax* RDTs specificity (99% [0.95CI: 99% to 100%]) [9].
	Repeatability (inter-operators)	*Kappa* > 0.8	*Kappa* > 0.9	Kappa statistic can be used to evaluate binary outcomes agreement. Suggested values are arbitrary.
	Reproducibility (inter-laboratories)	*Kappa* > 0.7	*Kappa* > 0.9	See *Repeatability*
**Operational aspects**	Assay format	End point, single-use *in vitro* diagnostic	End point single-use *in vitro* diagnostic	Suspected cases should be tested and treated as soon as possible and as such require a single determination per test.
	Assay throughput	Single assessment per test	Single assessment per test	See *assay format*
	Assay packaging	Package of single kits sharing reagents (if required) and user manual	Package of single kits with individual reagents sharing user manual	"M" and "O" reflect current packaging formats of RDTs.
	Operation conditions	5°C – 40°C<br>Up to 90% relative humidity (RH)	5°C – 45°C<br>Up to 90% RH	"M" and "O" reflect extreme conditions of endemic countries. RDT transportation and storage temperatures regularly exceed 30°C, rarely 40°C [10].
	Transportation and storage stability	≥ 12 months at 35°C and 70% RH with transport stress (3 days at 60 °C), no cold chain needed	≥ 12 months at 45°C and 90% RH with transport stress (3 days at 60 °C), no cold chain needed	"M" and "O" reflect typical and possible extreme transportation and storage conditions observed for RDTs [10].
	In use stability	> 1 hour for single-use test once opened	> 1 hour for single-use test once opened	Tests are likely to be used extemporaneously and as such this characteristic is unlikely to be limiting.
	Reagents reconstitution	Reconstitution of reagent acceptable if number of step is limited (≤ 5) and not requiring external equipment	All reagents provided and ready to use.	"M" is more stringent than the actual characteristic of LM (Giemsa solution preparation requires several precise steps).<br>"O" is met by current RDTs.
	Equipment	Small (≤ 100 cm^2 footprint) and portable (≤ 5 kg)	None	

Type	Characteristic	Minimal (M) / Description	Optimal (O)	Comment
	Power requirement	Battery operated with ≥ 24 hours testing autonomy	None	
	Maintenance	≤ once per year	None	
	Sample type	Capillary blood	Capillary blood or any less invasive validated sample	Sample types less invasive than capillary blood include saliva, urine, breath or transdermal detection [11].
	Sample volume	≤ 100 µL of capillary blood	≤ 50 µL of capillary blood	Variable for other sample types
	Sample preparation	≤ 5 steps	None	"M" reflects the actual characteristic of LM (*i.e.* fix, rinse, stain, rinse, dry). "O" is met by current RDTs.
	Overall test preparation	≤ 10 steps, of which ≤ 2 are timed	≤ 3 steps, of which ≤ 1 is timed	"M" and "O" reflect actual characteristics of LM and RDT.
	Time-to-result	≤ 1 hour	≤ 30 minutes	"M" is based on WHO recommendation, "O" would allow uninterrupted management between diagnostic and treatment. "M" is less stringent than malERA recommendations [1,12].
	Internal control	Included	Included	
	External control	Available	Included	External controls, such as positive control wells for RDT, are especially important in low endemic settings (*i.e.* in area of low positivity rate)
	Assay interpretation	Unequivocal, recorded by operator	Unequivocal, recorded by operator or electronically	
	Data capture	Manual by operator	Electronic automated	
	Data transfer	Manual by operator	Automated via internet or GSM connectivity	
	Training	≤ 1 day for inexperienced health worker	≤ 0.5 days for inexperienced health worker	Include plan for quality control and proficiency monitoring

Type	Characteristic	Minimal (M) / Description	Optimal (O)	Comment
	Biosafety	Class B IVD (moderate individual and low public health risk)	Class A IVD (low individual and public health risk)	According to risk-based classification of diagnostics for WHO prequalification [13].
	Language	English, Spanish and Portuguese	Local languages	
**Cost**	End user price per test	≤1.0 USD	≤ 0.5 USD	"O" is more stringent than malERA recommendation [1].
	Cost of diagnosis	≤ 2.0 USD	≤ 1.0 USD	RDT and LM costs of diagnosis were reported to be between 2.0 and 1.0 USD in 2011 in Uganda [14].

Supplementary References

1. The malERA Consultative Group on Diagnoses and Diagnostics. A Research Agenda for Malaria Eradication: Diagnoses and Diagnostics. PLoS Med. 2011;8: e1000396. doi:10.1371/journal.pmed.1000396.t001

2. World Health Organization. WHO Evidence Review Group on Malaria Diagnosis in Low Transmission Settings [Internet]. 21 Mar 2014 [cited 11 Oct 2015] pp. 1–33. Available: http://www.who.int/malaria/mpac/mpac_mar2014_diagnosis_low_transmission_settings_report.pdf

3. PATH. Target Product Profile: Point-of-Care Malaria Infection Detection Test. In: sites.path.org [Internet]. [cited 25 Jun 2016]. Available: http://sites.path.org/dx/files/2012/11/DIAMETER_IDT_TPP_FINAL_forwebsite.pdf

4. World Health Organization. Control and Elimination of Plasmodium Vivax Malaria. 2015.

5. FIND. Strategy for Malaria 2015-2020 [Internet]. 16 Sep 2015 [cited 6 Oct 2015] pp. 1–24. Available: http://www.finddiagnostics.org/export/sites/default/resource-centre/find_reports/pdfs/FIND_malaria_strategy_web_v03-2015.pdf

6. McKenzie FE, Jeffery GM, Collins WE. Plasmodium vivax blood-stage dynamics. J Parasitol. 2002;88: 521–535. doi:10.1645/0022-3395(2002)088[0521:PVBSD]2.0.CO;2

7. McKenzie FE, Jeffery GM, Collins WE. Gametocytemia and fever in human malaria infections. J Parasitol. 2007;93: 627–633. doi:10.1645/GE-1052R.1

8. Barber BE, William T, Grigg MJ, Parameswaran U, Piera KA, Price RN, et al. Parasite Biomass-Related Inflammation, Endothelial Activation, Microvascular Dysfunction and Disease Severity in Vivax Malaria. Stevenson MM, editor. PLoS Pathog. Public Library of Science; 2015;11: e1004558. doi:10.1371/journal.ppat.1004558

9. Abba K, Kirkham AJ, Olliaro PL, Deeks JJ, Donegan S, Garner P, et al. Rapid diagnostic tests for diagnosing uncomplicated non-falciparum or Plasmodium vivax malaria in endemic countries. Abba K, editor. Cochrane Database Syst Rev. Chichester, UK: John Wiley & Sons, Ltd; 2014;12: CD011431. doi:10.1002/14651858.CD011431

10. Albertini A, Lee E, Coulibaly SO, Sleshi M, Faye B, Mationg ML, et al. Malaria rapid diagnostic test transport and storage conditions in Burkina Faso, Senegal, Ethiopia and the Philippines. Malar J. BioMed Central Ltd; 2012;11: 406. doi:10.1186/1475-2875-11-406

11. Lukianova-Hleb EY, Campbell KM, Constantinou PE, Braam J, Olson JS, Ware RE, et al. Hemozoin-generated vapor nanobubbles for transdermal reagent- and needle-free detection of malaria. Proc Natl Acad Sci USA. 2014;111: 900–905. doi:10.1073/pnas.1316253111

12. World Health Organization. Guidelines for the Treatment of Malaria. 3rd ed. 2015 Apr pp. 1–318.

13. World Health Organization. Risk Based Classification of Diagnostics for WHO Prequalification. In: who.int [Internet]. 2014 [cited 7 Oct 2015]. Available: http://www.who.int/diagnostics_laboratory/evaluations/140513_who_risk_based_classification_of_ivds_for_pq_buffet.pdf?ua=1

14. Batwala V, Magnussen P, Hansen KS, Nuwaha F. Cost-effectiveness of malaria microscopy and rapid diagnostic tests versus presumptive diagnosis: implications for malaria control in Uganda. Malar J. BioMed Central Ltd; 2011;10: 372. doi:10.1186/1475-2875-10-372

15. Hofmann N, Mwingira F, Shekalaghe S, Robinson LJ, Mueller I, Felger I. Ultra-Sensitive Detection of Plasmodium falciparum by Amplification of Multi-Copy Subtelomeric Targets. Seidlein von L, editor. PLoS Med. Public Library of Science; 2015;12: e1001788. doi:10.1371/journal.pmed.1001788

16. Murphy SC, Prentice JL, Williamson K, Wallis CK, Fang FC, Fried M, et al. Real-time quantitative reverse transcription PCR for monitoring of blood-stage Plasmodium falciparum infections in malaria human challenge trials. Am J Trop Med Hyg. American Society of Tropical Medicine and Hygiene; 2012;86: 383–394. doi:10.4269/ajtmh.2012.10-0658
