# Supplementary material for: Defining the next generation of Plasmodium vivax diagnostic tests for control and elimination: Target product profiles
Source: PLoS Negl Trop Dis. 2017 Apr 3;11(4):e0005516. doi: 10.1371/journal.pntd.0005516 (PMC5391123; doi:10.1371/journal.pntd.0005516)
Supplement: S3 Table — (PDF) [file pntd.0005516.s003.pdf]

S3 Table. TPP PvB1: Point-of-care diagnosis of subpatent *Plasmodium vivax* infection

Type	Characteristic	Minimal (M) / Description	Optimal (O)	Comment
**Scope**	Intended use	The test goal is to provide an infection detection test for *P. vivax* able to detect a substantial fraction of all infections (symptomatic or asymtptomatic) for test-and-treat in any active infection detection interventions. Therefore, the test needs to accurately detect low density erythrocytic forms of *P. vivax* in a point-of-care manner.		
	Test outcome	Guide blood-stage and, if appropriate, liver-stage treatment in reactive and proactive case detection		
	Target population	The target population is any individual susceptible to suffer from a latent infection from *P. vivax*, including neonates, children, and pregnant women to enable treatment and, if appropriate, radical cure.		
	Target users	The target users include community health workers with minimal training and any health worker with a similar or superior training level.		
	Implementation level	The target implementation levels are community health facilities, health posts, health centers, district hospital as well as reference centers [5].		
**Performance**	Analytical sensitivity	Limit of detection for target analyte corresponding to a peripheral parasitaemia of 20 p/µL	Limit of detection for target analyte corresponding to a peripheral parasitaemia of 1 p/µL	"M" corresponds to a ten-fold increase in sensitivity as compared to RDTs and is line with malERA recommendation. "O" corresponds to a hundred-fold increase in sensitivity as compared to RDTs. For indirect tests (*e.g.* serology), the analytical sensitivity might not directly relate to parasitaemia.
	Analytical specificity	Discriminate between *P. vivax* and other *Plasmodium spp*. Do not cross-react with any other pathogen infecting humans	Discriminate between *P. vivax*, *P. falciparum* and other *Plasmodium spp*. Do not cross-react with any other pathogen infecting humans	"M" enables the specific identification of *P. vivax*. "O" provides a unique test for *P. vivax* and *P. falciparum* co-endemicity areas. Cross-reactivity between *P. vivax* and *P. ovale* might be beneficial to identify both of these relapsing species.
	Diagnostic outcome	Binary	Binary	A continuous (quantitative) outcome is not required for the intended use of the test.
	Diagnostic sensitivity	> 95% as compared to standard PCR with known limit of detection of 1 p/µL	≥ 99% as compared to standard PCR with known limit of detection of 1 p/µL	In line with malERA recommendations (but comparator not specified) [1]. Comparator might need to be adapted for indirect tests (*e.g.* based on serology).

Type	Characteristic	Minimal (M) / Description	Optimal (O)	Comment
	Diagnostic specificity	> 95% as compared to standard PCR with known limit of detection of 1 p/µL	≥ 99% as compared to standard PCR with known limit of detection of 1 p/µL	
	Repeatability (inter-operators)	*Kappa* > 0.8	*Kappa* > 0.9	Kappa statistic can be used to evaluate binary outcomes agreement. Suggested values are arbitrary.
	Reproducibility (inter-laboratories)	*Kappa* > 0.7	*Kappa* > 0.9	See *Repeatability*
**Operational aspects**	Assay format	End point, single-use *in vitro* diagnostic	End point single-use *in vitro* diagnostic	The assay format should enable the individual testing of 100 or less individuals in a point-of-care manner.
	Assay throughput	Single assessment per test	Single assessment per test, optional batch testing for higher throughput	See *assay format*
	Assay packaging	Package of single kits sharing reagents (if required) and user manual	Package of single kits with individual reagents sharing user manual	"M" and "O" reflect current packaging formats of RDTs.
	Operation conditions	5°C – 40°C<br>Up to 90% relative humidity (RH)	5°C – 45°C<br>Up to 90% RH	"M" and "O" reflect extreme conditions of endemic countries. RDT transportation and storage temperatures regularly exceed 30°C, rarely 40°C [10].
	Transportation and storage stability	≥ 12 months at 35°C and 70% RH with transport stress (3 days at 60 °C), no cold chain needed	≥ 12 months at 45°C and 90% RH with transport stress (3 days at 60 °C), no cold chain needed	"M" and "O" reflect typical and possible extreme transportation and storage conditions observed for RDTs [10].
	In use stability	> 30 minutes for single-use test once opened	> 2 hours for single-use test once opened	For batch testing, this characteristics is likely to impact the assay throughput.
	Reagents reconstitution	Reconstitution of reagent acceptable if number of step is limited (≤ 5) and not requiring external equipment	All reagents provided and ready to use.	"M" is more stringent than the actual characteristic of LM (Giemsa solution preparation requires several precise steps). "O" is met by current RDTs.
	Equipment	Small (≤ 100 cm^2 footprint) and portable (≤ 5 kg)	None	

Type	Characteristic	Minimal (M) / Description	Optimal (O)	Comment
	Power requirement	Battery operated with ≥ 24 hours testing autonomy	None	
	Maintenance	≤ once per year	None	
	Sample type	Capillary blood	Capillary blood or any less invasive validated sample	Sample types less invasive than capillary blood include saliva, urine, breath or transdermal detection [11].
	Sample volume	≤ 100 μL of capillary blood	≤ 50 μL of capillary blood	Variable for other sample types
	Sample preparation	≤ 5 steps	None	"M" reflects the actual characteristic of LM (*i.e.* fix, rinse, stain, rinse, dry). "O" is met by current RDTs.
	Overall test preparation	≤ 10 steps, of which ≤ 2 are timed	≤ 3 steps, of which ≤ 1 is timed	"M" and "O" reflect actual characteristics of LM and RDT.
	Time-to-result	≤ 30 minutes	≤ 10 minutes	Point-of-care testing requires short time-to-result values.
	Internal control	Included	Included	
	External control	Available	Included	External controls, such as positive control wells for RDT, are especially important in low endemic settings (*i.e.* in area of low positivity rate).
	Assay interpretation	Unequivocal, recorded by operator	Unequivocal, recorded by operator or electronically	
	Data capture	Manual by operator	Electronic automated	
	Data transfer	Manual by operator	Automated via internet or GSM connectivity	
	Training	≤ 5 days for inexperienced health worker	≤ 1 day for inexperienced health worker	Include plan for quality control and proficiency monitoring.
	Biosafety	Class B IVD (moderate individual and low public health risk)	Class A IVD (low individual and public health risk)	According to risk-based classification of diagnostics for WHO prequalification [13].

Type	Characteristic	Minimal (M) / Description	Optimal (O)	Comment
	Language	English, Spanish and Portuguese	Local languages	
**Cost**	End user price per test	≤2.0 USD	≤ 1.0 USD	“O” is more stringent than malERA recommendation [1].
	Cost of diagnosis	≤ 5.0 USD	≤ 2.0 USD	RDT and LM costs of diagnosis were reported to be between 2.0 and 1.0 USD in 2011 in Uganda [14].

Supplementary References

1. The malERA Consultative Group on Diagnoses and Diagnostics. A Research Agenda for Malaria Eradication: Diagnoses and Diagnostics. PLoS Med. 2011;8: e1000396. doi:10.1371/journal.pmed.1000396.t001

2. World Health Organization. WHO Evidence Review Group on Malaria Diagnosis in Low Transmission Settings [Internet]. 21 Mar 2014 [cited 11 Oct 2015] pp. 1–33. Available: http://www.who.int/malaria/mpac/mpac_mar2014_diagnosis_low_transmission_settings_report.pdf

3. PATH. Target Product Profile: Point-of-Care Malaria Infection Detection Test. In: sites.path.org [Internet]. [cited 25 Jun 2016]. Available: http://sites.path.org/dx/files/2012/11/DIAMETER_IDT_TPP_FINAL_forwebsite.pdf

4. World Health Organization. Control and Elimination of Plasmodium Vivax Malaria. 2015.

5. FIND. Strategy for Malaria 2015-2020 [Internet]. 16 Sep 2015 [cited 6 Oct 2015] pp. 1–24. Available: http://www.finddiagnostics.org/export/sites/default/resource-centre/find_reports/pdfs/FIND_malaria_strategy_web_v03-2015.pdf

6. McKenzie FE, Jeffery GM, Collins WE. Plasmodium vivax blood-stage dynamics. J Parasitol. 2002;88: 521–535. doi:10.1645/0022-3395(2002)088[0521:PVBSD]2.0.CO;2

7. McKenzie FE, Jeffery GM, Collins WE. Gametocytemia and fever in human malaria infections. J Parasitol. 2007;93: 627–633. doi:10.1645/GE-1052R.1

8. Barber BE, William T, Grigg MJ, Parameswaran U, Piera KA, Price RN, et al. Parasite Biomass-Related Inflammation, Endothelial Activation, Microvascular Dysfunction and Disease Severity in Vivax Malaria. Stevenson MM, editor. PLoS Pathog. Public Library of Science; 2015;11: e1004558. doi:10.1371/journal.ppat.1004558

9. Abba K, Kirkham AJ, Olliaro PL, Deeks JJ, Donegan S, Garner P, et al. Rapid diagnostic tests for diagnosing uncomplicated non-falciparum or Plasmodium vivax malaria in endemic countries. Abba K, editor. Cochrane Database Syst Rev. Chichester, UK: John Wiley & Sons, Ltd; 2014;12: CD011431. doi:10.1002/14651858.CD011431

10. Albertini A, Lee E, Coulibaly SO, Sleshi M, Faye B, Mationg ML, et al. Malaria rapid diagnostic test transport and storage conditions in Burkina Faso, Senegal, Ethiopia and the Philippines. Malar J. BioMed Central Ltd; 2012;11: 406. doi:10.1186/1475-2875-11-406

11. Lukianova-Hleb EY, Campbell KM, Constantinou PE, Braam J, Olson JS, Ware RE, et al. Hemozoin-generated vapor nanobubbles for transdermal reagent- and needle-free detection of malaria. Proc Natl Acad Sci USA. 2014;111: 900–905. doi:10.1073/pnas.1316253111

12. World Health Organization. Guidelines for the Treatment of Malaria. 3rd ed. 2015 Apr pp. 1–318.

13. World Health Organization. Risk Based Classification of Diagnostics for WHO Prequalification. In: who.int [Internet]. 2014 [cited 7 Oct 2015]. Available: http://www.who.int/diagnostics_laboratory/evaluations/140513_who_risk_based_classification_of_ivds_for_pq_buffet.pdf?ua=1

14. Batwala V, Magnussen P, Hansen KS, Nuwaha F. Cost-effectiveness of malaria microscopy and rapid diagnostic tests versus presumptive diagnosis: implications for malaria control in Uganda. Malar J. BioMed Central Ltd; 2011;10: 372. doi:10.1186/1475-2875-10-372

15. Hofmann N, Mwingira F, Shekalaghe S, Robinson LJ, Mueller I, Felger I. Ultra-Sensitive Detection of Plasmodium falciparum by Amplification of Multi-Copy Subtelomeric Targets. Seidlein von L, editor. PLoS Med. Public Library of Science; 2015;12: e1001788. doi:10.1371/journal.pmed.1001788

16. Murphy SC, Prentice JL, Williamson K, Wallis CK, Fang FC, Fried M, et al. Real-time quantitative reverse transcription PCR for monitoring of blood-stage Plasmodium falciparum infections in malaria human challenge trials. Am J Trop Med Hyg. American Society of Tropical Medicine and Hygiene; 2012;86: 383–394. doi:10.4269/ajtmh.2012.10-0658
