# Supplementary material for: Defining the next generation of Plasmodium vivax diagnostic tests for control and elimination: Target product profiles
Source: PLoS Negl Trop Dis. 2017 Apr 3;11(4):e0005516. doi: 10.1371/journal.pntd.0005516 (PMC5391123; doi:10.1371/journal.pntd.0005516)
Supplement: S4 Table — (PDF) [file pntd.0005516.s004.pdf]

S4 Table. TPP PvB2: Population screening for *Plasmodium vivax* infection surveillance

Type	Characteristic	Minimal (M) / Description	Optimal (O)	Comment
**Scope**	Intended use	The test goal is to provide indication of current or recent *P. vivax* infection for epidemiological surveys and surveillance activities not necessarily linked with a direct treatment of positive cases. Therefore, the test needs to accurately detect biomarkers of recent infection or low density erythrocytic forms of *P. vivax* with a high throughput and analytical sensitivity.		
	Test outcome	Inform monitoring efforts to guide response interventions		
	Target population	The target population is any individual susceptible to suffer from a latent infection from *P. vivax*, including children, and pregnant women.		
	Target users	The target users are laboratory operators with a substantial proficiency in laboratory work.		
	Implementation level	The target implementation levels are district hospital as well as reference laboratories [5].		
**Performance**	Analytical sensitivity	Limit of detection for target analyte corresponding to a peripheral parasitaemia of 0.1 p/µL	Limit of detection for target analyte corresponding to a peripheral parasitaemia of 0.01 p/µL	"O" corresponds to a two-fold improvement compared to current state-of-the art technologies [15,16].<br>For indirect tests (*e.g.* serology), the analytical sensitivity might not directly relate to parasitaemia.
	Analytical specificity	Discriminate between *P. vivax* and other *Plasmodium spp*. Do not cross-react with any other pathogen infecting humans	Identify all *Plasmodium spp.* and discriminate species. Do not cross-react with any other pathogen infecting humans	A high level of specificity and information content is expected from a specialized laboratory assay.
	Diagnostic outcome	Test-dependant	Test-dependant	A quantitative outcome (parasitaemia level) would be nice-to-have for tests detecting current infections. For indirect test (*e.g.* serology), the outcome might be associated with probability of past infection.
	Diagnostic sensitivity	> 95% as compared to a validated standard with an analytical sensitivity at least equal to the index test	≥ 99% as compared to a validated standard with an analytical sensitivity at least equal to the index test	In line with malERA recommendations (but comparator not specified) [1]. Comparator might need to be adapted for indirect tests (*e.g.* based on serology).

Type	Characteristic	Minimal (M) / Description	Optimal (O)	Comment
	Diagnostic specificity	> 90% as compared to a validated standard with an analytical sensitivity at least equal to the index test	≥ 95% as compared to a validated standard with an analytical sensitivity at least equal to the index test	Comparator might need to be adapted for indirect tests (*e.g.* based on serology).
	Repeatability (inter-operators)	*Kappa* > 0.8	*Kappa* > 0.9	Kappa statistic can be used to evaluate binary outcomes agreement. Suggested values are arbitrary.
	Reproducibility (inter-laboratories)	*Kappa* > 0.7	*Kappa* > 0.9	See *Repeatability*
**Operational aspects**	Assay format	96-well format assay	384-well format assay or higher	
	Assay throughput	Batch testing in line with assay format	Batch testing in line with assay format	
	Assay packaging	Package of assay-specific components and user manual	Package of all assay components and user manual	
	Operation conditions	15°C – 30°C<br>Up to 60% relative humidity (RH)	15°C – 35°C<br>Up to 90% RH	"M" and "O" reflect expected controlled laboratory conditions in endemic countries [10].
	Transportation and storage stability	≥ 6 months at ≤4°C and 60% RH, transport at ≤4°C acceptable	≥ 12 months at 30°C and 90% RH with transport stress (3 days at 60 °C), no cold chain needed	"M" reflects laboratory conditions that can be relatively easily achieved in endemic countries.
	In use stability	> 30 minutes	> 1 hour	For batch testing, this characteristics is likely to impact the assay throughput.
	Reagents reconstitution	Reconstitution of reagent acceptable	All reagents provided and ready to use.	
	Equipment	Transportable (≤ 20 kg)	Portable (≤ 5 kg)	

Type	Characteristic	Minimal (M) / Description	Optimal (O)	Comment
	Power requirement	Power supply, if needed, adapted for the voltage type found in *P. vivax* endemic countries	Battery operated with ≥ 24 hours testing autonomy	
	Maintenance	≤ once per year	None	
	Sample type	Capillary blood	Capillary blood or any less invasive validated sample	Sample types less invasive than capillary blood include saliva, urine, breath or transdermal detection [11].
	Sample volume	≤ 200 µL of capillary blood	≤ 100 µL of capillary blood	The analytical sensitivity is directly linked with the total volume of sample assessed. Volumes might vary for other sample types than capillary blood.
	Sample preparation	≤ 5 steps	None	Complex sample preparation is acceptable if it does not impact the overall assay throughput.
	Overall test preparation	≤ 20 steps, of which ≤ 5 are timed	≤ 10 steps, of which ≤ 2 are timed	Complex assay procedure is acceptable if it does not impact the overall assay throughput.
	Time-to-result	≤ 1 month	≤ 7 days	
	Internal control	Included	Included	
	External control	Available	Included	
	Assay interpretation	Unequivocal, recorded electronically	Identical to “M”	
	Data capture	Electronic, automated	Identical to “M”	
	Data transfer	Manual by operator	Automated via internet or GSM connectivity	
	Training	≤ 2 weeks for health worker with a substantial proficiency in laboratory work	≤ 1 week for health worker with a substantial proficiency in laboratory work	Include plan for quality control and proficiency monitoring.

Type	Characteristic	Minimal (M) / Description	Optimal (O)	Comment
	Biosafety	No reagent associated with acute toxicity hazards	No reagent associated with health hazards	According to the Globally Harmonized System of Classification and Labelling of Chemicals (GHS).
	Language	English, Spanish and Portuguese	Local languages	
**Cost**	End user price per test	≤1.0 USD	≤ 0.1 USD	The high throughput batch testing should facilitate a low test price.
	Cost of diagnosis	≤ 1.2 USD	≤ 0.5 USD	See End user price per test.

Supplementary References

1. The malERA Consultative Group on Diagnoses and Diagnostics. A Research Agenda for Malaria Eradication: Diagnoses and Diagnostics. PLoS Med. 2011;8: e1000396. doi:10.1371/journal.pmed.1000396.t001

2. World Health Organization. WHO Evidence Review Group on Malaria Diagnosis in Low Transmission Settings [Internet]. 21 Mar 2014 [cited 11 Oct 2015] pp. 1–33. Available: http://www.who.int/malaria/mpac/mpac_mar2014_diagnosis_low_transmission_settings_report.pdf

3. PATH. Target Product Profile: Point-of-Care Malaria Infection Detection Test. In: sites.path.org [Internet]. [cited 25 Jun 2016]. Available: http://sites.path.org/dx/files/2012/11/DIAMETER_IDT_TPP_FINAL_forwebsite.pdf

4. World Health Organization. Control and Elimination of Plasmodium Vivax Malaria. 2015.

5. FIND. Strategy for Malaria 2015-2020 [Internet]. 16 Sep 2015 [cited 6 Oct 2015] pp. 1–24. Available: http://www.finddiagnostics.org/export/sites/default/resource-centre/find_reports/pdfs/FIND_malaria_strategy_web_v03-2015.pdf

6. McKenzie FE, Jeffery GM, Collins WE. Plasmodium vivax blood-stage dynamics. J Parasitol. 2002;88: 521–535. doi:10.1645/0022-3395(2002)088[0521:PVBSD]2.0.CO;2

7. McKenzie FE, Jeffery GM, Collins WE. Gametocytemia and fever in human malaria infections. J Parasitol. 2007;93: 627–633. doi:10.1645/GE-1052R.1

8. Barber BE, William T, Grigg MJ, Parameswaran U, Piera KA, Price RN, et al. Parasite Biomass-Related Inflammation, Endothelial Activation, Microvascular Dysfunction and Disease Severity in Vivax Malaria. Stevenson MM, editor. PLoS Pathog. Public Library of Science; 2015;11: e1004558. doi:10.1371/journal.ppat.1004558

9. Abba K, Kirkham AJ, Olliaro PL, Deeks JJ, Donegan S, Garner P, et al. Rapid diagnostic tests for diagnosing uncomplicated non-falciparum or Plasmodium vivax malaria in endemic countries. Abba K, editor. Cochrane Database Syst Rev. Chichester, UK: John Wiley & Sons, Ltd; 2014;12: CD011431. doi:10.1002/14651858.CD011431

10. Albertini A, Lee E, Coulibaly SO, Sleshi M, Faye B, Mationg ML, et al. Malaria rapid diagnostic test transport and storage conditions in Burkina Faso, Senegal, Ethiopia and the Philippines. Malar J. BioMed Central Ltd; 2012;11: 406. doi:10.1186/1475-2875-11-406

11. Lukianova-Hleb EY, Campbell KM, Constantinou PE, Braam J, Olson JS, Ware RE, et al. Hemozoin-generated vapor nanobubbles for transdermal reagent- and needle-free detection of malaria. Proc Natl Acad Sci USA. 2014;111: 900–905. doi:10.1073/pnas.1316253111

12. World Health Organization. Guidelines for the Treatment of Malaria. 3rd ed. 2015 Apr pp. 1–318.

13. World Health Organization. Risk Based Classification of Diagnostics for WHO Prequalification. In: who.int [Internet]. 2014 [cited 7 Oct 2015]. Available: http://www.who.int/diagnostics_laboratory/evaluations/140513_who_risk_based_classification_of_ivds_for_pq_buffet.pdf?ua=1

14. Batwala V, Magnussen P, Hansen KS, Nuwaha F. Cost-effectiveness of malaria microscopy and rapid diagnostic tests versus presumptive diagnosis: implications for malaria control in Uganda. Malar J. BioMed Central Ltd; 2011;10: 372. doi:10.1186/1475-2875-10-372

15. Hofmann N, Mwingira F, Shekalaghe S, Robinson LJ, Mueller I, Felger I. Ultra-Sensitive Detection of Plasmodium falciparum by Amplification of Multi-Copy Subtelomeric Targets. Seidlein von L, editor. PLoS Med. Public Library of Science; 2015;12: e1001788. doi:10.1371/journal.pmed.1001788

16. Murphy SC, Prentice JL, Williamson K, Wallis CK, Fang FC, Fried M, et al. Real-time quantitative reverse transcription PCR for monitoring of blood-stage Plasmodium falciparum infections in malaria human challenge trials. Am J Trop Med Hyg. American Society of Tropical Medicine and Hygiene; 2012;86: 383–394. doi:10.4269/ajtmh.2012.10-0658
